# Case-Based Discussion in United Kingdom General Practice Training: A Critical Analysis

**DOI:** 10.7759/cureus.13166

**Published:** 2021-02-06

**Authors:** Lubna Rauf

**Affiliations:** 1 Clinical Education, Qatar College of Medicine, Doha, QAT

**Keywords:** family medicine, uk gp training, formative assessments, case based discussion

## Abstract

Case-based discussion (CbD) is a form of workplace-based assessment to assess the progress of learning in general practice trainees in the United Kingdom. We aim to identify the need and rationale behind CbD. The usefulness of CbD in the trainee’s learning will be analyzed with the help of well-recognized parameter such as utility equation. It will also be considered whether the assessment delivers what it is supposed to in actual practice. The current pitfalls will be identified with suggestions for potential improvements.

## Introduction and background

Case-based discussions (CbDs) are primarily a form of formative assessment in general practice (GP) training [1]. Formative assessment looks at learning progression through feedback and informs the GP trainees about their performance. It is a reflective process that intends to promote the trainee’s attainment of competencies required to become a fully trained GP [2]. In CbD, GP trainees choose a case from their own clinical practice and present it to the trainer. The trainee will also highlight the aspects of the case that they would like to discuss. The discussion allows the trainer to grade the trainee’s competence on a template included in the trainee’s portfolio [3].

The trainees are encouraged to reflect on their performance and point out what was done well and what proved to be a challenge. The discussion allows a retrospective review of the performance in relation to a particular case. The standard against which the trainee is judged is always at the level of competence expected of a doctor who is certified to practice independently as a GP. As Kare-Silver and Mehay comment, trainees at the start of their training struggle with the concept of an assessment checking their competencies set out for a fully trained GP [2]. But they explain that by doing this: the trainees at the start of their training will gain the knowledge and insight about their starting point and will show progression toward competence in years to come through this process. CbD identifies strengths and weaknesses throughout the training period and enables development. It results in a developmental feedback and objective goals to enhance progression [2]. This style of formative assessment is directly in line with the concept of constructive alignment described by Biggs [4]. He stated that the components in the teaching system, especially teaching methods and assessment tasks, should be aligned with the learning activities, which, in turn, produce competencies according to the intended learning outcomes. The curriculum is mapped in assessments such as CbDs, which helps make holistic judgments about the attainment of intended learning outcomes. These are sought for qualities of performance, and it is these that need to be stated clearly so that the trainee’s actual performance can be judged against those qualities [4].

Background of CbD

The curriculum document for GP trainees in 2005 highlighted the need for change in the assessment methods used in the Membership Exam of the Royal College of General Practitioners (MRCGP) [5]. The alignment of learning activities and assessments with intended learning outcomes was absent. The training system failed to monitor a trainee’s progress due to a lack of formative assessments. Most of the assessment methods used were summative and did not have any relationship to the trainees’ actual work environment. Rethans et al. had already highlighted that what doctors do in a controlled setting is far away from what they do in their actual practice [6]. It also failed to identify trainees with potential deficiencies. It was recognized by the Royal College of General Practitioners (RCGP) that all pre-existing forms of assessment in GP training were not in line with the General Medical Council’s (GMC) principles of good medical practice or standards set out by the regulatory body. The need for balance between formative and summative assessments was highlighted [5]. The new curriculum introduced a training system involving formative and summative assessments throughout the training period to increase the authenticity of the training process, thus resulting in better trained GPs [5].

The principles of a good workplace-based assessment (WPBA) were discussed by Swanwick and Chana [7]. They proposed some key features that WPBAs should have, naming that these should be competency-based, developmental, evidential, locally accessed, and triangulated. The CbDs were seen as a potential tool of professional development. Their origin comes from chart-stimulated recall (CSR) [8]. As pointed out by Norcini, CSR was developed by Maatesch for use by the American Board of Emergency Medicine. Single clinical encounters were used to assess competence in various areas of clinical care. Although the CSR was quite different in its design and usage, it provided the basic framework to develop CbDs to be used in the United Kingdom (UK) GP training.

## Review

Utility of case-based discussion

From the discussion so far, CbD can be considered as an assessment that supports the idea of formative assessment and integrates learning by becoming a part of learning and not an extra bolt-on at the end of the whole process [9]. Van der Vleuten researched and developed the principles that measure the usefulness of an assessment and termed those as utility [10]. The utility of an assessment has been defined as a product of reliability, validity, cost-effectiveness, educational impact, and acceptability, and named as the Utility Equation. It can be expressed as follows:

Utility = Reliability x Validity x Educational impact x Cost-effectiveness x Acceptability

The Utility Equation has been used as an authentic and accepted yardstick for any assessment method by PMETB (Postgraduate Medical Education and Training Board) when it laid out the principles of a good assessment system in 2007 [11]. This guide describes the Utility Equation as an excellent framework for assessment design and evaluation. The discussion about each element of the utility equation will further highlight the fact whether the CbD is a useful assessment used in MRCGP or whether it needs modification or reconfiguration in relation to its functions as a tool of learning and assessment of progression.

Reliability

Reliability is a term pertaining to the reproducibility of results or outcomes of an assessment [1,12]. Reliability of a test can be measured using repeated testing and configuring reproducibility of the scores achieved as a coefficient, which can be from 0 to 1. A score of 1 means perfect reliability. Van Der Vleuten and Schuwirth considered the coefficient of 0.80 to be a minimum acceptable standard for an assessment. They also pointed out that no assessment system is entirely reliable or totally unreliable. A fairly subjective and non-standardized assessment method can be highly reliable if used in an appropriate context [12]. According to Williamson and Osborne, CbDs are only moderately reliable [13]. They argue that the competencies assessed by CbDs are wide-ranging and subjective, making results challenging to objectify. This notion is contrary to what was said by Van der Vleuten et al., as they did not see quantification of an examinee’s performance as a barrier in reliability and instead considered subjective assessments equally reliable if used appropriately. However, a reliance on an assessor’s ability leaves a marked gap, making CbDs less reliable. Also, as Kare-Silver and Mehay indicated, trainer calibration is usually poor and the CbD criteria are not universally understood, thus making CbDs less reliable [2]. As cited in an electronic learning module designed by London Deanery on WPBAs, several issues challenge the reliability of CbDs. As assessor’s ability in carrying out a CbD can be quite variable, which will result in variation in results irrespective of the trainee’s performance, a fact called “inter-observation variation”. Similarly, a “good day, bad day” phenomenon can interfere with the trainee’s actual performance, which is called “intra-observer variation”. Also, the factor of a trainee getting variable grades along the same attributes in different CbDs can occur from one case to another due to no apparent reason, which is termed “case specificity” [12].

On a satisfactory note, Etheridge and Boursicot found CbDs to have good reliability and were able to differentiate between doctors in good standing and the ones who were poorly performing if compared to other assessment methods [14]. Therefore, to ensure that CbDs become a reliable form of assessment, the training of trainers is paramount. The calibration of the standards is vital and needs periodic review and evaluation, with the trainee involvement in the whole process [2].

Validity

The term “validity” in the context of utility of assessment is defined as the extent to which a competence in an assessment is actually being assessed [1]. It cannot be assumed that one CbD on its own can determine whether a trainee is competent in a certain domain. A trainer can extract a wealth of knowledge about the trainee’s actual performance against a pre-set criteria [13] with the help of a CbD. But as Schuwirth LWT, van der Vleuten explain, validity is a very specific term and is a direct measure of the criterion [1]. Therefore, for the CbD to be valid, it needs to measure exactly what it is meant for. Therefore, many CbDs will need to be conducted by more than one trainer to make them more valid.

Cost-effectiveness

It seems that CbDs are conducted in a GP setting free of any cost and are hence an ideal form of assessment, but this is somewhat controversial. Etheridge and Boursicot pointed out that evidence regarding the true cost of CbD is scarce [14]. In actual practice, however, it takes around an hour to complete a CbD from discussion to feedback and logging it on to an electronic portfolio. This time is obviously deducted from the working hours of the trainer and impacts the practice budget in terms of replacing that clinical time by a locum doctor. The deanery pays the practice every month to cover the trainee’s salary and the cost of training. Assessments such as CbDs require more one-to-one time of the trainee with the trainer. The prospect of income generation through training GPs is getting narrower every day. It may have implications to de-motivate practices from the actual process of training and also discourage potential GP trainers. Likewise, it can have implications on the quality of assessments done by the trainers and trainees’ demoralization.

Educational impact

Educational impact seems to be the most important factor that can be discussed in relation to the CbDs. Van der Vleuten and Schuwirth have demonstrated the wide-ranging acceptability of a strong linkage between WPBAs and their educational impact [12]. They also accepted that there is a lack of evidence to prove that link that accepts assessments such as CbD as a tool for learning. They highlighted the fact that maybe this impact has not been studied in detail because it is challenging to collect information about various related factors. Factors that influence the educational impact of an assessment are described by Schuwirth and van der Vleuten and are called content, format, scheduling of the assessment, and the regulatory structure of the assessment program [1].

The content of CbD is designed to mirror various elements of curriculum in UK GP training [5]. The blueprinting of curriculum through WPBAs can prove to have a high level of educational impact through feedback that drives future learning [11]. Also, the topics chosen by the trainees and their difficulty levels can be linked to the educational impact of the assessment.

The format of a CbD in GP training is based on one-to-one discussion between a trainee and a trainer. The trainees are expected to describe the clinical encounter in their words, reflecting on their thought process and what they find challenging. Therefore, it is a process of being self-critical and reflective with an experienced trainee, which can help to align the thought process to enhance future learning [15].

Scheduling or timing is somewhat arbitrary and opportunistic while carrying out CbDs in actual practice. The utility and effectiveness of CbDs can be increased to a greater degree if these are well spaced out during the training time and are carried out when there are fewer pressures such as summative assessments of MRCGP part one and part two.

The discussion about the educational impact of WPBAs by Schuwirth and van der Vleuten highlighted the fact that the widely accepted notion of students learning mostly what they would be assessed for is not something to point out negatively but can be utilized to maximize the potential of a formative assessment to direct the trainee towards learning that they ought to learn [1].

Acceptability

Acceptability is a multifaceted attribute of an assessment. For an assessment to continue effectively, it has to be acceptable by all stakeholders; for instance, the trainees, trainers, deaneries, RCGP, and GMC. The Royal Colleges can strive to design a perfect assessment in line with GMC guidance and the RCGP training curriculum, but if it is not acceptable or practical enough for trainees and trainers, it will become a useless tool. CbD has been accepted and relied upon for years by the RCGP and GMC to be one of the most effective tools to map a trainee’s performance and progress. When combined with other assessments, it produces a comprehensive outlook to the trainee’s journey in a training program [2]. Etheridge and Boursicot have stated that the research so far shows some evidence that trainees are finding this shift in assessments hard to accept [14]. They postulate that this lack of flexibility on the trainees’ behalf might be due to the lack of clarity of CbDs to be a formative or summative tool of assessment, which, sometimes, can prove confusing for the trainers as well.

Strengths and limitations

CbD appears to be a reasonable form of WPBA. However, it has limitations such as any other form of assessment. In a survey carried out by Bodgener and Tavabie, views of GP trainees and trainers were collected in relation to the effectiveness of the CbD [16]. The study showed that the majority of stakeholders do not see it as a true and total reflection of performance. It is important to highlight the most important factor of a CbD, which has the maximum educational impact. In a study carried out by Mehta et al., the process of feedback through CbD has been shown to be the main driver for its educational impact [17]. In that study, the pediatric trainees were asked to express their views about the educational impact of CbD specifically in relation to feedback. Trainees valued the educational impact of CbD through the process of reflection. Feedback was found more useful from assessors who carried it out positively and had more training to conduct WPBAs. Time constraints and less suitable environment were thought to have a negative educational impact. The choice of more challenging cases was expressed to have better educational impact. In a systemic review carried out by Miller and Archer, CbDs fail to show any impact on a trainee’s performance. However, CbDs and other WPBAs demonstrated a positive educational impact on subjective reports [18].

In a guide given in 2010 by GMC to implement WPBAs, certain strengths and limitations of WPBAs were identified [19]. That evidence, in the context of our discussion specifically for CbDs, can be utilized due to its direct relevance. Strengths include a high potential for validity. CbDs are mainly trainee-led and map achievement in the competency framework. CbDs provide feedback and encourage a nurturing culture. CbDs can help to identify trainees with difficulties early on during the training and can provide a sample of performance across a wide area of the curriculum. Limitations outlined are that these assessments cannot be reliable on their own and need further evidence for reassurance. Aspiration to excellence can be lost if trainers and trainees start to use CbDs as a tickbox exercise. Due to a lack of proper understanding by the trainer and trainee, it can become opportunistic and efficacy can be lost. WPBAs require time and training. The inexperience of the trainer can lead to the improper execution of the whole process, making it less effective and reliable.

Suggestions for improvement

The purpose and intention of WPBA should be to maximize educational impact. The trainee and the trainer should strive towards excellence throughout the training. The progression from bare minimum that is acceptable to becoming an expert should be demonstrable from the whole process. If CbDs in the first year of training are showing a "meets expectations" grading, it should not stay the same in final year and should show progression in terms of improvement towards “above expectations” or “fit for licensing”. The trainees usually choose the cases where they have done well for CbDs instead of cases where they struggle. There seems to be a generalized expectation from trainees that they should come in category of “meets expectations” from the start, which makes the whole point of validity questionable [2]. If the trainees are made aware of the fact that this is not a “pass” or “fail” exercise, they might be able to choose cases to maximize learning and not just to score points. Also, to maximize learning, they might choose to spread them out with frequent gaps instead of cramping them all together towards the end [9]. The whole process should be open and transparent and should be evaluated throughout the training. It is best done if “triangulated” [20]. This term refers to the process of gathering evidence of progression and competency from more than one assessor on more than one occasion and by using more than one assessment method.

## Conclusions

The feedback is the most important aspect of the CbDs. The feedback should be on the performance and not the behavior. It should include examples and must include suggestions for improvement. The training of the trainers is of paramount importance in the whole process of CbD in specific and WPBAs in general. Lack of training leads to a poor execution of the CbD. Maximum benefit from a WPBA is gained when it is accompanied by skilled and expert feedback.
